# Parents’ Beliefs About the Benefits and Detriments of Mobile Screen Technologies for Their Young Children’s Learning: A Focus on Diverse Latine Mothers and Fathers

**DOI:** 10.3389/fpsyg.2020.570712

**Published:** 2020-10-09

**Authors:** Wendy Ochoa, Stephanie M. Reich

**Affiliations:** ^1^Tufts University, Medford, MA, United States; ^2^School of Education, University of California, Irvine, Irvine, CA, United States

**Keywords:** mobile, screens, children, parents, beliefs, Latino, technology, attitudes

## Abstract

Young children’s use of mobile screens is increasing despite the American Academy of Pediatrics’ recommendations to limit screen use. Research on TV has found that maternal beliefs about the effects of screens on children’s learning and parental socioeconomic status influence children’s media consumption. However, few studies have explored parents’ beliefs about mobile screens and whether there are differences in beliefs by socioeconomic status, particularly within the largest ethnic minoritized group — Latines. Because Latines are a socioeconomically and linguistically heterogenous group, but are often represented by low-income mothers in research, it is important to understand whether there are socioeconomic and linguistic differences on how and why Latine mothers *AND* fathers permit their children to use mobile screens. This study used in-depth, semi-structured interviews to understand how and why Latine mothers (low-income = 10, middle-to-high income = 10) and fathers (low-income = 10, middle-to-high income = 10) permitted their children (0–4 years) to use mobile screens. Specifically, we discussed their beliefs about how mobile screens support and hinder their children’s learning and how their children used them. Results from qualitative content analysis showed that mothers and fathers, across income, education levels, and language use, believed that they, as parents, were the key decision-makers in determining the extent to which mobile screens supported and hindered their young children’s learning. They described mediation strategies of selecting appropriate content, setting time limits, and monitoring use, to ensure that their children primarily benefited from device use. However, two distinctions were noted. Parents with a high school diploma or beyond stressed the importance of co-using devices with their children. This was not mentioned by less formally educated parents. Additionally, low-income parents with diverse educational levels, mentioned the importance of continuously monitoring device use to avoid their children encountering inappropriate content. Findings can inform work seeking to promote optimal media habits among Latine families.

## Introduction

Mobile screen technologies, such as smartphones and tablets, have permeated the everyday lives of most US families with young children across socioeconomic and ethnic groups, including Latine^1^ families (Kabali et al., 2015; Common Sense Media, 2017; Pew Research Center, 2017). In fact, as of 2017, 98% of families with children under the age of 8 years own at least one smartphone and 78% own at least one tablet device (Common Sense Media, 2017). Although higher-income families are still slightly more likely than lower-income families to have access to high speed internet (96% vs. 74%), low-income and higher-income families are just as likely to own a smartphone and have children who own a personal tablet device (Common Sense Media, 2017). Importantly, recent reports show that children as young as 6 months old are increasingly being exposed to more time viewing or using mobile screen devices, especially children from low-income homes (Kabali et al., 2015; Common Sense Media, 2017).

The upward trend in children’s exposure to mobile screen devices is of particular interest because extensive research on TV and emerging studies on mobile screens have found positive and negative associations between children’s exposure to screens and key child outcomes, depending on how the devices are used (Mendelsohn et al., 2010; Roseberry et al., 2013; Zack and Barr, 2016; Madigan et al., 2019). For example, a study conducted among a majority of low-income, Latina mothers (94%) showed that verbal interactions between mothers and their 14-month-old toddler while using screen media moderated the negative impact of media exposure on children’s language outcomes (Mendelsohn et al., 2010). That is, media use was negatively associated with children’s language outcomes only in the absence of mother-child interactions while viewing or using screens (Mendelsohn et al., 2010). More recently, a study conducted by Zack and Barr (2016) among predominantly White, middle-class mothers and their toddlers showed that children were able to transfer what they learned from a 2D touchscreen device to a 3D object when their mothers scaffolded them during the task (e.g., modeling device use, talk).

Due to the prevalence of mobile screen devices and the implications these could have on young children’s outcomes, it is important that we not only investigate how young children are using them, but that we also understand factors that might contribute to patterns of device use in diverse families. From the extensive research, primarily conducted in the context of TV, it is well known that maternal beliefs about the role of screens on children’s learning and parents’ socioeconomic status (SES) are two of the factors that most consistently predict children’s screen consumption (Certain and Kahn, 2002; Njoroge et al., 2013; Rideout, 2014). To date, however, very few studies have focused on exploring parents’ views about mobile screen technologies, and the few that do exist have primarily consisted of survey studies.

It is important to explore parents’ views about mobile screens because mobile devices are nearly ubiquitous in the lives of children. Further, their portability and multifunctional capabilities not only permit families to use them in the same way they would use a TV (e.g., watch a show), but provide a host of other uses, such as instant access to the Internet, interactive apps/games, and video chat – all possible in any place and at any time. Additionally, the touchscreen interface enables very young children to successfully use these devices, even without assistance from an adult. Such constant connectivity and affordances could shape parents’ beliefs about mobile screens and their role in their child’s learning differently from their beliefs about TV, and also contribute to differences in the ways families are taking advantage of these features and allowing their children to use them. However, because most studies investigating parents’ beliefs about mobile screens have consisted of surveys, our understanding of their beliefs about the role of mobile screens on their children’s learning have been limited to the questions posed by researchers, who are also likely from the majority culture. Hence, we likely do not yet have a complete understanding about how and why minoritized parents might believe mobile screen technologies are beneficial or detrimental to their young children’s learning. Moreover, despite the prevalence of mobile screens among socioeconomically and ethnically diverse families, White, middle-class parents make up the vast majority of samples in the extant research. In most of these studies, ethnic minority parents, particularly Latines, have comprised only a small portion of the entire sample, been disproportionately represented by low-income households, or their socioeconomic status has not been stated (Rideout, 2014; Wartella et al., 2014; Radesky et al., 2016; Common Sense Media, 2017; Sergi et al., 2017; McCloskey et al., 2018). As a result, it has been difficult to discern whether the SES or ethnic differences found in parent beliefs are associated with SES or cultural differences for ethnic minority parents (Cabrera and The SRCD Ethnic and Racial Issues Committee, 2013). Thus, we have virtually no understanding about the role of SES on parents’ beliefs about their young children’s use of mobile screen technologies among ethnically minoritized parents.

A particular ethnic group who has been largely excluded from research on screen media are Latine parents, even though they are the largest minoritized ethnic group in the United States, and are among the ethnic groups most likely to rely on smartphones for access to the Internet (Pew Research Center, 2017). Further, within Latine parents, fathers and Spanish-speaking parents have been especially underrepresented in screen media research. Not including fathers in media research among Latines is limiting because two out of three Latine children live in a two-parent household (Pew Research Center, 2015), and research has shown that fathers make unique and important contributions to their children’s development (Cabrera et al., 2007). Furthermore, it is important to include linguistically diverse Latine parents because research in various areas of parenting has shown that English- and Spanish-speaking parents sometimes differ in their ideas about parenting practices and child development, likely due to nativity (Keels, 2009). Therefore, to address some of the gaps in the literature, this study focuses on obtaining a deeper understanding of socioeconomically and linguistically diverse Latine mothers and fathers’ beliefs about the role of mobile screen devices on their very young children’s learning. Parents of children ages 0–4 years old are the focus of this study because this is the age when screens (e.g., TV) have been found to have a large impact on children’s developmental outcomes (Rice et al., 1990; Mendelsohn et al., 2010). Additionally, this is also an age when parents can play a major role in regulating the content and amount of time children spend viewing or using screens, which can shape the child’s media consumption trajectory.

## Parent Beliefs About the Role of Mobile Screen Technologies on Children’s Learning

Although research exploring parents’ beliefs about the role of mobile screen technologies on their children’s learning is still in its early stages, emerging research suggest that most parents believe mobile screen technologies could both support and detract from their children’s learning (Wartella et al., 2014; Radesky et al., 2016; Common Sense Media, 2017; Sergi et al., 2017; McCloskey et al., 2018). For example, a national survey conducted by Wartella et al. (2014) among a socioeconomically diverse sample of predominantly White (56%), Latine (23%), and Black (9%) parents of children 8 years old and younger found that 37% of the parents believed mobile screen technologies had a positive effect on their children’s math skills and creativity. However, 46% of these parents also believed that mobile devices negatively affected their children’s attention span (Wartella et al., 2014). Similar results were found in a qualitative study among five highly educated, racially diverse parents of children ages 4–7 years old. Specifically, most of these parents thought that mobile screen technologies had helped their children improve their math and language skills (Sergi et al., 2017). Nevertheless, most parents also expressed concerns about their children’s excessive use of mobile devices, the random pop-up advertisements, unlimited access to entertainment apps, and the possibility that their children would become socially isolated due to excessive device use (Sergi et al., 2017). Many of these same concerns were also voiced by US parents in a recent national survey study (Common Sense Media, 2017).

Although only a handful of studies have made SES and ethnic comparisons of parents’ beliefs about the role of mobile screen technologies on their children’s learning, interesting differences have been found within these studies. For instance, through the use of semi-structured interviews among socioeconomically diverse White (58%), Black (29%), and Latine (5%) mothers (74%) and fathers (26%) of children between the ages of 0 and 8 years old, Radesky et al. (2016) found that more low-income parents than middle-to-high income parents reported feeling good about exposing their children to mobile screen devices, because they believed they would give their child an advantage later on in life. Similarly, a national survey of a socioeconomically diverse sample of Latine (43%), White (39%), and Black (18%) parents of children ages 2–10 years found that low-income parents tended to attribute more educational benefits to mobile devices than middle-to-high income parents (Rideout, 2014). Within this study, the author also found that Latine and African American parents were more likely than White parents to consider mobile screen devices to be an important source of learning for their children (Rideout, 2014).

In contrast to the aforementioned findings, however, a more recent national survey among a socioeconomically diverse sample of White (56%), Latine (22%), and Black (10%) mothers (60%) and fathers (40%) of children 8 years and younger found that Latine parents tended to express more concerns about the effects of mobile screen technologies on their children than White and Black parents (Common Sense Media, 2017). Moreover, Latine parents were more likely than parents from other ethnic groups to agree with the statement that the less time children spent with media the better (Common Sense Media, 2017). These latter findings align with those found in a recent survey study among a low-income sample of primarily Latine (78%) mothers (86%) and fathers (6%) of children in Head Start Centers (age 4 years) (McCloskey et al., 2018). Findings from this study showed that Latine parents were less likely than parents from other ethnic groups to say that their children used mobile screen technologies to learn. Across these few studies, it is unclear whether Latine parents hold more positive, negative, or neutral views than other ethnic groups about the role of mobile screen technologies on their children’s learning. Moreover, we have almost no understanding on how SES and language might influence the beliefs of Latine parents, and especially Latino fathers, of young children because most studies have consisted of surveys with low-income Latine mothers. Therefore, the aim of this qualitative study is to use semi-structured interviews to understand how a sample (*n* = 40) of socioeconomically and linguistically diverse Latine mothers and fathers of children 4 years old or younger believe mobile screen technologies support and/or hinder their children’s learning.

## Materials and Methods

### Study Design

A semi-structured interview design was used to obtain a deeper understanding of how and why diverse Latine parents of young children permit their children to use mobile screens, along with their beliefs about how these devices contribute or detract from children’s learning. Qualitative approaches are appropriate to use when the goal is to identify and understand different perspectives about a given phenomenon (Giacomini and Cook, 2000).

### Recruitment and Participants

#### Recruitment

Latine mothers and fathers of children under the age of five, living in Southern California were invited to participate in one-on-one interviews between June 2018 and April 2019. Recruitment was done in one of three ways. The first method involved asking participants who were interested but ineligible to participate in another research study if they would be interested in participating in this study instead. The second method included posting flyers at businesses, churches, and grocery stores. Finally, the third method was through snowball sampling. All parents were asked to participate in a 45–60-min audio-recorded, in-person interview in English or Spanish in a place of their choice, including their home or a local coffee shop. To be eligible to participate in the study, parents needed to report 1) owning at least one mobile screen device and having access to the Internet on it, 2) self-identify as Latine, and 3) have at least one child who was 4 years old or younger. No restrictions were placed on the parents’ age, number of children, marital status, nationality, or primary language spoken.

Given that we were interested in understanding the role of SES and parent gender on Latine parents’ use and beliefs about mobile devices, we wanted to ensure equal representation of socioeconomically diverse Latine mothers and fathers. Therefore, using the approach of previous researchers, we used income as a proxy for SES and recruited parents from low-income and middle-to-high income households (Davis-Kean, 2005; Bodnar-Deren et al., 2017). Parents’ income was determined by calculating their poverty index using the following demographic information: (1) total household annual income, (2) total number of people living in their household at least 4 days of the week, and (3) the number of these individuals who were minors and adults. In total, we purposefully recruited 20 low-income parents (10 mothers and 10 fathers) and 20 middle-to-high income parents (10 mothers and 10 fathers). Although we initially aimed to recruit equal numbers of low-income and middle-to-high income Spanish-speaking mothers and fathers, we were not successful because all monolingual Spanish-speaking parents who expressed interest in participating in our study had low incomes and all middle-to-high income parents were fluent in English (and all but one father reported speaking Spanish too). Therefore, Spanish-only-speaking parents are not represented in the middle-to-high income groups.

#### Participants

In total, 40 Latine parents (*n* = 20 mothers, *n* = 20 fathers) of children between the ages of 0–4 years of age participated. All mothers and fathers were distributed equally across the low-income (*n* = 20) and middle-to-high income (*n* = 20) groups. There were a total of four couples, two in each income group. The next sections describe the demographic characteristics of parents from each of the four groups.

#### Low-Income Mothers

Low-income mothers (*n* = 10) ranged in age from 22 to 36 years (*M* = 28.77, *SD* = 5.33). On average, they had two children (*M* = 2.1, *SD* = 1.37) and all had at least one child under the age of 5 years (*M* = 1.9 yrs., *SD* = 1.5). Of the target children that were 4 years old or younger, 21% were female and 79% were male. Sixty percent of the mothers had a high school education or less, 20% had some college, and 20% obtained a Bachelor’s degree. More than half of the mothers (60%) were born outside of the United States, with most being from Mexico (50%) or Ecuador (10%). These mothers had been in the United States for an average of 15 years (*M* = 15.08, *SD* = 9.19). Finally, the majority of mothers (70%) were English-Spanish bilingual and only 30% were monolingual Spanish-speakers. Noticeably, two monolingual Spanish-speaking mothers had an elementary school education and one had a Bachelor’s degree.

#### Low-Income Fathers

Low-income fathers (*n* = 10) ranged in age from 26 to 45 years (*M* = 31.50, *SD* = 6.28). On average they had two children (*M* = 1.9, *SD* = 0.87) and all had at least one child under the age of 5 years (*M* = 2.0 yrs., *SD* = 1.2). Of the target children (i.e., 0–4 years), 36% were female and 64% male. Forty percent of the fathers had a high school education or less, 50% had completed some college or a 2-year degree, and 10% obtained a Bachelor’s degree. Additionally, 40% of the fathers were born in Mexico and had been in the United States for an average of 24 years (*M* = 24.14, *SD* = 14.45). Finally, the majority of fathers (70%) were English-Spanish bilingual and only 30% were monolingual Spanish-speakers. Noticeably, the two monolingual Spanish-speaking fathers had a middle-school education or less.

#### Middle-to-High Income Mothers

Middle-to-high income mothers (*n* = 10) ranged in age from 23 to 35 years (*M* = 30.60, *SD* = 4.74). On average they had one child (*M* = 1.20, *SD* = 0.42) and all had a child under the age of 5 years (*M* = 2.0 yrs., *SD* = 1.3). Of the target children (i.e., 0–4 years), 33% were female and 67% male. Twenty percent of the mothers had completed some college, 20% had a Bachelor’s degree, and 60% had a Master’s degree or beyond. Additionally, all but one of the mothers were born in the United States. Finally, all mothers were English-Spanish bilinguals.

#### Middle-to-High Income Fathers

Middle-to-high income fathers (*n* = 10) ranged in age from 23 to 41 years (*M* = 33.50, *SD* = 5.72). On average they had two children (*M* = 1.90, *SD* = 0.99) and all had at least one child under the age of 5 years (*M* = 2.3 yrs., *SD* = 1.4). Of the target children (i.e., 0–4 years), 38% were female and 62% male. Sixty percent of the fathers had completed some college or a 2-year degree and 40% had a Master’s degree or beyond. Additionally, only one of the fathers was born in Mexico and had been in the United States for 20 years. Finally, 90% of the fathers were English-Spanish bilinguals and only one was an English-monolingual speaker.

### Procedure

Once interested parents were confirmed to be eligible to participate in the study, a date and time was set to interview the parent. At the start of the interview, the first author, an English-Spanish bilingual Latina, provided the parent with an informed consent form. The form included a description and goals of the study, the parent’s right to stop the interview at any time or to opt to not answer any question that made them uncomfortable, and asked for permission to audio-record the interview. All parents were assured that their confidentiality would be protected. After the parent signed the informed consent form, the researcher turned on an audio-recording device and began the interview. When the interview was over, parents were compensated with a $10 Target gift card and a bilingual children’s book. A university Institutional Review Board (IRB) approved all procedures and materials.

### Measures

#### Parents’ Income Category

Parents were asked to report on their: (1) total household annual income, (2) total number of people living in their household at least 4 days of the week, and (3) the number of these individuals who were minors and adults. Using this information, parental income level was determined by calculating their poverty index, which compared a family’s annual household income to an income threshold level that varied by family size and composition (i.e., number of children and adults).

The threshold levels are updated every year for inflation with the Consumer Price Index (United States Census Bureau, Poverty Thresholds, 2016). A family is considered to be living in poverty if their household annual income is less than the threshold level (United States Census Bureau, Poverty Thresholds, 2016). In their study, Brooks-Gunn et al. (1999) identified five income-to-needs ratio: (1) deep poverty (income-to-needs ratio less than 0.50), (2) poverty (income-to-needs ratio greater than or equal to 0.50, but less than 1.0), (3) near poverty (income-to-needs ratio between 1.0 and 1.5), low income (income-to-needs ratio between 1.5 and 2.0) and middle income (income-to-needs ratio greater than or equal to 2.0). However, we only identified two categories for this study: Low income (income-to-needs ratio: less than 2.0) and middle-to-high income (income-to-needs ratio: equal to or greater than 2.0).

#### Background Questionnaire

Parents were asked to answer a 15-item background questionnaire created for this study. Questions included whether they were a mother or father, their and their children’s gender, ages, ethnicity, family income, number of people living in their household, their education level, marital status, nationality, years living in the United States, and language(s) spoken.

#### Semi-Structured Interview

A Spanish and English semi-structured, in-depth interview with open-ended questions was created for this study. The semi-structured interview asked parents about their beliefs and attitudes about the ways mobile screen devices support and/or hinder their children’s learning (e.g., *Do you think smartphones and/or tablets have benefited your children’s learning? How? Do you think smartphones and or tablets can be bad for your child’s learning, how?*). Parents were also asked about the types of device limits they set for their children (e.g., *Do you have specific time limits for your children to use mobile devices? What kind of limits? Why?*) and to also describe how the target child used mobile screen devices (e.g., *What does your child typically do when s/he uses the smartphone and/or tablet?*). It is important to highlight that in answering the questions, parents were asked to think about their child(ren) that were 4 years of age or younger. To ensure that the questions were clear and interpreted as intended in both languages, extensive Spanish and English cognitive interviews were done with other low and middle-to-high income parents in this geographic region prior to data collection.

### Qualitative Coding and Analysis

The audio-recordings were transcribed in their original language. The transcripts were then coded in their original language using the MAXQDA qualitative software. All parents were given a pseudonym to protect their identity. Qualitative content analysis (Schreier, 2014) was then employed with a blended approach to answer our main research questions. Open coding enabled the emergence of new themes (Strauss and Corbin, 1998) while deductive coding guided the coding of data in light of existing findings (Saldaña, 2003). For instance, we expected to see key aspects of parental monitoring (deductive codes were framed in the literature) but did not have expectations about parents’ views of benefits and detriments globally (inductive codes were derived from patterns that emerged from the data). By sorting parents’ own words conceptually (Strauss and Corbin, 1990), we identified patterns of practices and beliefs about the ways mobile screens benefit and hinder their children’s learning.

Our initial coding did not consider gender or whether parents had low or middle-to-high incomes. Instead, patterns were identified across interviews as soon as they were transcribed. The main categories were generated through a concept-driven strategy that used a combination of prior research, logic, everyday knowledge, and the main research questions (i.e., benefits and detriments to children’s learning) by the lead researcher. This resulted in the generation of three main categories (i.e., parents’ beliefs about the ways mobile screen devices benefit children’s learning, parents’ beliefs about the ways mobile screen devices hinder children’s learning, and the ways parents regulated their children’s use of mobile screens). Sub-categories were then generated within each of these larger main categories using the data-driven strategy of subsumption summarizing to capture the *specific ways* in which parents believed mobile screens were beneficial (e.g., learning concepts) and detrimental (e.g., lack of social interactions) to children’s learning, along with the specific ways in which parents regulated their children’s use of these technologies. After the lead researcher developed the main categories and subcategories, a group of independent researchers were asked to read relevant excerpts of one of four random interviews that the lead researcher had coded to create their own main categories and sub-categories. Then, they met to compare their categories and subcategories. Categories and sub-categories were compared and discussed, and the most frequently created main categories and subcategories were then used to develop the final coding frame. Upon finalizing the coding frame, all interviews were coded using the coding frame. Then, a subset of excerpts of the transcripts were shared with five doctoral students who were asked to code into the coding frame or recommend different codes, if needed.

To ensure data trustworthiness, not only did the lead researcher hold peer debriefing meetings with other researchers but also often asked interviewees if her interpretation of what they had said during the interview was correct. Finally, after all transcripts had been coded using the final coding scheme, the researchers used the features of MAXQDA software to obtain frequencies and make SES, gender, and linguistic comparisons to answer the research question. A 2:1 ratio was used to determine whether there were differences in themes and sub-themes on income and parent gender. However, because there were only six monolingual Spanish-speakers and one monolingual English-speaker compared to 33 English-Spanish bilingual parents, we only considered there to be differences between monolingual Spanish-speakers and English-Spanish bilinguals when none of the six Spanish-speakers mentioned a particular theme or sub-theme but 30% or more of the 33 English-Spanish bilinguals did. Similarly, if none of the English-Spanish bilinguals mentioned a theme but the six monolingual Spanish-speakers did, we also considered there to be a difference between linguistic groups. Because there was only one monolingual English-speaker, no differences or similarities with monolingual Spanish-speakers or English-Spanish bilinguals were discussed.

## Results

Descriptive analyses indicated that the majority of parents across income and gender had access to the Internet and also owned about the same number of mobile screen technologies. Specifically, households across income (*M* = 2.95 low, *M* = 2.53 middle-to-high income, *p*ns) and gender (*M* = 2.68 mothers, *M* = 2.79 fathers, *p*ns) groups had access to two smartphones per household on average. Similarly, no differences were found in the average number of tablets owned per household across income (*M* = 0.95 low, *M* = 1.10 middle-to-high income, *p*ns) or gender (*M* = 0.95 mothers, *M* = 1.10 fathers, *p*ns) groups. Unexpectedly, a slightly higher percentage of target children (ages 0–4) from low-income households (37%) owned a personal tablet device than children from middle-to-high income households (16%). Additionally, most households across income (70% low, 80% middle-to-high income) and gender (70% mothers, 80% fathers) groups had access to both home Wi-Fi and data through their smartphones (Income: 85% low, 95% middle-to-high; Parent gender: 95% mothers, 85% fathers).

Analyses of the transcripts revealed that four themes emerged that centered around parents’ beliefs about the ways mobile screen devices benefited their children’s learning. Two themes captured parents’ discussions about what they believed their children could learn from mobile screen devices (e.g., academic concepts, language), and the two other themes captured the specific activities they believed their child should do on the device to support their learning (e.g., view videos, use apps/games). Three themes also emerged that revolved around parents’ beliefs about the ways mobile screen devices could hinder their children’s learning (i.e., lack of social interactions, dependence, and accessing inappropriate content). However, in addition to the specific benefits and hindrances parents associated with mobile screen technologies, one major theme emerged that centered on how parents regulated children’s use, which we refer to as *mediation practices*. This included such sub-themes as ensuring the content and/or game used was appropriate for the child, restricting the amount of time that the child was permitted to use the device, constantly monitoring the child while using the device to ensure they did not deviate into inappropriate content, and co-using the device with the child to scaffold their learning. Importantly, across income, gender, and linguistic groups, most parents believed that mobile screen technologies could both support and hinder children’s learning depending on the types of mediation practices parents implemented. Hence, parental mediation practices, as a theme, focused on parents’ opinions about the specific types of mediation practices (each sub-theme practice) they believed contributed to mobile devices being beneficial or detrimental toward children’s learning. Comparisons across income groups (i.e., low and middle-to-high income), education level, parent gender, and language groups are described in more detail for each theme and subtheme below. Additionally, comparisons across groups are summarized in Table 1.

**TABLE 1:** Comparison of themes and subthemes across groups.

Themes and Sub-themes	Total % theme mentioned (*n* = 40)	Differences in themes by groups								
		Parent gender		Income		Language			Education	Differences
Benefits	97%	Mothers (*n* = 20)	Fathers (*n* = 20)	Low-Income (*n* = 20)	Middle-High Income (*n* = 20)	Monolingual Spanish-Speaking (*n* = 6)	Bilingual (*n* = 33)	Monolingual English-speaking (*n* = 1)		
Child learns academic concepts	67%	16	11	13	14	5	21	1	Elementary-Ph.D.	*No difference between groups*
		*No difference*		*No difference*		*No difference*			*No difference*	
Child develops language skills	87%	15	20	17	18	6	28	1	Elementary-Ph.D.	*No difference between groups*
		No difference		No difference		No difference			No difference	
Child learns from videos	77%	15	16	16	15	5	25	1	Elementary-Ph.D.	*No difference between groups*
		*No difference*		*No difference*		*No difference*			*No difference*	
Child learns from apps	40%	6	10	9	7	3	12	1	Elementary-Ph.D.	*No difference between groups*
		*No difference*		*No difference*		*No difference*			*No difference*	
**Detriments**	92%									
Social interactions	35%	7	7	4	10	0	14	0	High School-Ph.D.	*Differences across 3 groups*
		*No difference*		*Difference*		*Difference*			*Difference*	
Dependence or addiction	45%	9	9	7	11	0	18	0	High School-Ph.D.	*Difference for 1 group*
		*No difference*		*No difference*		*Difference*			*No Difference*	
Accessing inappropriate content	30%	6	6	6	6	2	9	1	Middle School-MA	*No difference between groups*
		*No difference*		*No difference*		*No difference*			*No difference*	
**Mediation strategies**	85%	18	16	17	17	6	27	1	Elementary-Ph.D.	*No difference between groups*
		*No difference*		*No difference*		*No difference*			*No difference*	
*Quality content*	67%	14	13	12	15	5	21	1	Elementary-Ph.D.	*No difference between groups*
		*No difference*		*No difference*		*No difference*			*No difference*	
*Time limits*	40%	9	7	8	8	5	10	1	Elementary-Ph.D.	*No difference between groups*
		*No difference*		*No difference*		*No difference*			*No difference*	
*Parental monitoring*	22%	3	6	6	3	1	7	1	Elementary-BA	*Difference across 2 group*
		*Difference*		*Difference*		*No difference*			*No difference*	
*Co-use*	20%	5	4	4	5	2	6	1	High School-Ph.D.	*Difference for 1 group*
		*No difference*		*No difference*		*No difference*			*Difference*	
**Mediation combination**	52%	12	9	9	12	4	16	1	Elementary-Ph.D.	*No difference between groups*
		*No difference*		*No difference*		*No difference*			*No difference*	

### Benefits of Mobile Screen Technologies on Children’s Learning

Virtually all parents (*n* = 39) believed that mobile screen technologies could offer some benefit to their children’s learning. Although we left the term “children’s learning” vague during our questioning, parents generally described learning from a device as learning some type of academic concept (e.g., colors, numbers, shapes, animals, letters) promoting Spanish or English language learning, and, from watching videos or using apps/playing games. Though the majority of parents thought children learned from viewing videos on the device, a little less than half of the sample also thought young children could learn from using apps/playing games. The next few sections describe these themes in more detail.

#### Learning Concepts

In total, 67% of parents across income (*n* = 13 low, *n* = 14 middle-to-high income), gender (*n* = 16 mothers, *n* = 11 fathers), linguistic groups (*n* = 5 monolingual Spanish-speaking, *n* = 21 English-Spanish bilinguals, *n* = 1 monolingual English-speaking), and education levels (elementary school to Ph.D.) thought their children could learn academic concepts through the use of mobile screen devices, with most parents mentioning numbers, letters, shapes, patterns, body parts, animals, food labels, and music. For example, Eric, a middle-to-high income, English-Spanish bilingual father who completed some college, described how his child had learned how to count through using the smartphone, “*she knows how to count to ten through like*… *what is that guy called? Bleepy? And then she tries to count to twenty. It’s a little, you know, but it’s funny*… *it’s cute.*” A little over half (55%) of the academic concepts that parents discussed their child had learned were attributed to videos. For instance, Carlos, a middle-to-high income English-Spanish bilingual father with a master’s degree discussed that his children had learned how to count from watching videos, “*there’s these videos. To me they’re weird but like a lot of what he watches is educational. So they’ll have all the marvel characters. All these super heroes, and then the super heroes are like*…*they teach him how to count.”* Additionally, 45% of the concepts that children had learned were also attributed to the use of educational apps/games. For example, Aniceto, a low-income Spanish-speaking father with an elementary education discussed how his children had learned letters from apps/games, “*usualmente, hay unos juegos que mencionan el nombre de la letra y basado a eso es como ellos pueden aprender.*” [English translation: *“usually, there are some games that say the name of the letter and based on that is how they can learn.”*].

#### Learning Language Skills

In addition to learning academic concepts, 87% of parents across income (*n* = 17 low, *n* = 18 middle-to-high income), gender (*n* = 15 mothers, *n* = 20 fathers), linguistic groups (*n* = 6 Spanish-speaking, *n* = 28 English-Spanish bilinguals, *n* = 1 monolingual English-speaking), and education levels (elementary school to Ph.D.) also thought children could learn language skills, such as develop their English or Spanish skills, from using mobile screen devices. As Karina, a low-income English-Spanish bilingual mother with a high school degree explained, “*yeah it has [benefited child]. She learned how to speak English from there. Cuz I wasn’t speaking English to her at all, so she’s learning, and now she speaks English and Spanish to me. She says the colors in English and Spanish to me.*” The majority (67%) of language skills that parents discussed their children had learned were attributed to videos. For example, Daniel, a low-income English-Spanish bilingual father with a 2-year certificate, discussed that his child had learned different languages from watching Youtube videos, “*I mean, there’s a lot of Youtubers. A lot of people that, you know, are from different ethnicities that speak other languages, you know? And he [child] tends to copy them sometimes.”* Additionally, 33% of the language skills that parents mentioned their child had learned were attributed to apps/games. For instance, Anthony, a low-income English-Spanish bilingual father who had completed some college described his child’s use of an app to learn Spanish and English words, “*so they can translate*… *cuz I remember, I remember I had an app for a while they [children] would play with, and it would like say in English and Spanish like certain things, like apple, manzana, and stuff like that.”*

#### Viewing Videos to Learn

Most parents (77%) said their children could learn concepts and/or language skills by viewing videos on the device, and no differences existed by income (*n* = 16 low, *n* = 15 middle-to-high income), gender (*n* = 15 mothers, *n* = 16 fathers), linguistic groups (*n* = 5 monolingual Spanish-speaking, *n* = 25 English-Spanish bilinguals, *n* = 1 monolingual English-speaking), or education level (elementary school to Ph.D.). Additionally, many parents of children ages 3 months to 4 years thought their children’s learning could benefit from viewing videos on the device. For example, Cindy, a middle-to-high income English-Spanish bilingual mother with a Master’s degree, said, “*they could learn a new language. Um. one of the things I want her to do is, I want her to learn English and Spanish. So like I talk to her in Spanish and I try to put like, when I have the phone, nursery rhymes in Spanish*… *So I go on Youtube and that’s mainly how I’ve used it. I would say I use it every day*.” Table 2 contains a list of the specific types of videos parents across income and parent gender groups said their children viewed.

**TABLE 2:** Specific video platforms, programs, and topics parents said their children viewed.

Content of videos	Low-income mothers	Low- income fathers	Middle- high income mothers	Middle-high income fathers	Totals
Cartoons	4	3	2	2	11
Teen Titans		1			1
The Magic School Bus				1	1
Sesame Street/Elmo	1		1	2	4
Colors			1		1
Numbers			1	1	2
Shapes			1	1	2
Spanish songs/lullabies	3	1	3	1	8
ABCs			2	1	3
Animals	1	1		1	3
Potty-training			1		1
Fitness	1				1
Children’s toy reviews		1		3	4
Making slime	1				1
Playing with playdough	1		1	1	3
Other children playing			2		2
Family videos	3	2	3	3	11
Lullabies	3		4	5	12
Netflix	1	1			2
Total	29	10	21	20	

#### Using Apps to Learn

Almost half of parents (40%) thought their children could learn academic concepts and/or language skills from using apps. These parents were distributed across income (*n* = 9 low, *n* = 7 middle-to-high), parent gender (*n* = 6 mothers, *n* = 10 fathers), linguistic groups (*n* = 3 monolingual Spanish-speaking, *n* = 12 English-Spanish bilingual, 1 = monolingual English-speaking) and education levels (elementary school to Ph.D.). For example, Ricardo, a middle-to-high income English-Spanish bilingual father with a Ph.D., expressed his opinion on whether mobile screen technologies could benefit his children’s learning, “*I think definitely with um*…*there’s a lot of good apps that um*… *teach kids um*…*how to recognize letters, you know? And how to sound out words with the letters. Um*…*I think there’s a lot of good educational apps for kids*.” Furthermore, while neither of the two couples who had infants thought their children could learn from apps at their very young age, many parents of children ages 1.5–4 years thought their children could learn from using apps. Table 3 contains a list of the specific types of apps parents across income and parent gender groups said their children used or viewed.

**TABLE 3:** Specific types of apps parents said their children used.

App type/Name for the child	Low-income mothers	Low- income fathers	Middle- high income mothers	Middle-high income fathers	Totals
Patterns, puzzles, and maps	1	1	2		4
Coloring and art	1	1	4		6
Colors		2	1		3
Animals		2		1	3
Letters and reading	1	1	5	2	9
Spanish		1	1		2
Music		1	1	2	4
Numbers and math	1	1	2	1	5
Shapes		2	1	1	4
Entertainment game	2	1	1	3	6
Unsure		1			1
Total	6	14	18	10	

### Detriments of Mobile Screen Technologies on Children’s Learning

In addition to thinking that mobile screen technologies could benefit children’s learning, almost all parents (92%) also thought these devices could be detrimental to children’s learning. However, parents’ descriptions of “learning” when discussing detriments associated with mobile screen technologies encompassed such things as lack of social interactions for the child, children’s dependence on or addiction to the device, and encountering inappropriate content. Although the concerns of children’s dependence on or addiction to the device and encountering inappropriate content were mentioned across all gender, income, and educational groups, being worried about children’s social interactions was a concern that was more prevalent among middle-to-high income parents than low-income parents. The next few sections discuss the themes related to hindrances in greater detail.

#### Lack of Social Interactions

In total, 35% of parents expressed concerns about how mobile screen technologies could be detrimental to their children’s social interactions. For example, Yaritza, a middle-to-high-income English-Spanish bilingual mother with a Master’s degree, said, “*I still feel like, yes the technology and everything is great in her age but I feel like it does. it can interfere with it in terms of social interactions or them wanting to go out and be social and wanting them to go out and play.*” Noticeably however, slightly more middle-to-high income parents (*n* = 5 mothers, *n* = 5 fathers) than low-income parents (*n* = 2 mothers, *n* = 2 fathers) expressed concerns about the negative effects mobile screen technologies could have on their children’s social interactions. Furthermore, none of the monolingual Spanish-speaking parents expressed this concern, but two of the six Spanish-speaking parents (education level: high school – bachelor’s degree) had an infant and might not have experienced this issue yet. However, there might still be differences by language that should be further explored in future studies. In looking at the education level of the four monolingual Spanish-speaking parents who had children older than 1-year-old, the highest level of formal education attained was some high school. Thus, overall, it appears that education of a high school degree or higher was associated with worrying about mobile screen technologies interfering with children’s social interactions.

#### Dependence on or Addiction to the Device

A large percentage of parents (45%) also worried about the possibility of their children becoming dependent on, or addicted to, the mobile device. For instance, Yvonne, a low-income English-Spanish bilingual mother who completed some college, described, “*when he wants to go to sleep, he will just grab my phone and demand that I put something for him. That’s the downside. I don’t want him to like get addicted to it. And I don’t know he just sees it as, as something that he has to be on all the time now*.” This concern was spread across income (*n* = 7 low, *n* = 11 middle-to-high income) and gender (*n* = 9 mothers, *n* = 9 fathers) groups, but not linguistic groups. That is, none of the six monolingual Spanish-speaking parents (education level: elementary – bachelor’s degree) expressed the concern that their child could become dependent or addicted to the mobile device. This suggests that this concern might be more prevalent among parents of children older than one who have a high school degree or more than among parents with lower levels of formal education. It could also reflect differences in access to information about technology dependence based on language.

#### Accessing Inappropriate Content

In addition to sharing concerns about children’s lack of social interactions and dependence on the device, 30% of parents also expressed concern that their child would come across inappropriate content while using the device. This concern was dispersed across income (*n* = 6 low, *n* = 6 middle-to-high income), gender (*n* = 6 mothers, *n* = 6 fathers), linguistic groups (*n* = 2 monolingual Spanish-speaking, *n* = 9 English-Spanish bilinguals, 1 = monolingual English-speaking), and education levels (middle school – Master’s degree). For example, Gerardo, a low-income English-Spanish bilingual father who completed a 2-year certificate, captured the anxiety of many parents when he said, “*like advertisements or there’s this one program. I don’t know if it’s still there anymore. I know there was a lot of complaints from parents, cuz I saw it on the news as well, that it was on some show where it’s like Spider Man and Anna from Frozen and um*… *they did some things that are not like meant for children*.” Most of the parents who expressed concerns about their children coming across inappropriate content also reported monitoring their children’s use of the device. However, these parents felt that they had little control over the random ads that suddenly appeared when their child was viewing a video or video links their children would click on when they turned their attention away. It should be noted that half of the parents that brought up this issue (*n* = 6) had a high school degree or beyond and used content restrictions (e.g., parental controls) on the device.

### Importance of Parental Mediation Practices

Although most parents believed that mobile screen devices both benefited and hindered their children’s learning, the vast majority of parents (85%) across income (*n* = 17 low, *n* = 17 middle-to-high income), gender (*n* = 18 mothers, *n* = 16 fathers), linguistic groups (*n* = 6 monolingual Spanish-speaking, *n* = 27 English-Spanish bilingual, 1 = monolingual English-speaking), and education levels (elementary school to Ph.D.) also discussed their important role, as parents, in determining the extent to which mobile screen technologies could support and limit their child’s learning. As Luis, a middle-to-high income English-Spanish bilingual father with a Master’s degree explained, “*it’s gotta be hand in hand with um, what the parent is doing.*” Parents’ descriptions of mediation strategies included the importance of appropriate content or apps, setting time limits, monitoring children’s activities with the device, and assisting or helping the child understand the content encountered when using the device (i.e., co-use). However, although all of the aforementioned mediation strategies were cited, some were mentioned more frequently than others.

#### Quality Considerations of Content of Video or App

Across income (*n* = 12 low, *n* = 15 middle-to-high income), gender (*n* = 14 mothers, *n* = 13 fathers), linguistic groups (*n* = 5 monolingual Spanish-speaking, *n* = 21 English-Spanish bilingual, 1 = monolingual English-speaking), and education levels (elementary school to Ph.D.), most parents (67%) talked about the importance of ensuring children were viewing “appropriate” or “educational” content in videos and or apps. When prompted, most parents described “educational” content as videos or apps that taught children specific academic concepts, such as numbers, colors, shapes, or language, such as letter sounds or Spanish/English vocabulary. Olga, a low-income English-Spanish bilingual mother with a high school degree, stressed the importance of ensuring the content was age-appropriate when she stated, “*um*…*que no tengan mucha violencia para su edad. Y que sean entretenidos, que sean adecuados a la edad de el niño.*” [English translation: “*um*…*that they [videos/apps] don’t have a lot of violence for his age. And that are entertaining and appropriate for the child”*].

#### Setting Time Limits

The second most frequently mentioned mediation strategy by parents across income (*n* = 8 low, *n* = 8 middle-to-high income), gender (*n* = 9 mothers, *n* = 7 fathers), linguistic groups (*n* = 5 monolingual Spanish-speaking, *n* = 10 English-Spanish bilingual, 1 = monolingual English-speaking), and education levels (elementary school to Ph.D.) was setting time limits for children when they used mobile screen technologies (40%). Parents saw setting time limits as a way to maximize the learning benefits of the device while minimizing its detriments. For example, Chayo, a low-income monolingual Spanish-speaking mother with an elementary school education, gave the following response when asked if she thought mobile devices could benefit her children’s learning “*creo que*… *les ayudaría un poco pero no tanto. Creo que cierta*…*media hora*… *um*… *pero no demasiado tiempo. Si les serviría un poco.*” [English translation: “*I think that*…*it would help them a little bit but not a lot. I think that certain*…*half an hour*…*um*… *but not too much time. It would help them a little bit*”]. Similarly, most parents also mentioned limiting the amount of time or the frequency of device use by their child. For example, Jennifer, a middle-to-high income English-Spanish bilingual mother who had completed some college stated, “*we’re not specific with minutes but we try to not go more than like 30 or 35 min.”*

#### Parental Monitoring

The third type of mediation strategy that was mentioned by 22% of parents across groups was monitoring their children’s use of mobile devices. Parental monitoring was often described as the importance of constantly checking or knowing what children were doing on the mobile device without necessarily co-using the device with children. For this category, slightly more low-income parents (education level: elementary school- bachelor’s degree) (*n* = 6) than middle-to-high income parents (*n* = 3) talked about the importance of parental monitoring. Additionally, more fathers (*n* = 6) than mothers (*n* = 3) also mentioned this strategy. Nevertheless, parental monitoring was mentioned by Spanish- and English-speaking parents (*n* = 1 monolingual Spanish-speaking, *n* = 7 English-Spanish bilingual, 1 = monolingual English-speaking). Olga, a low-income English-Spanish bilingual mother with a high school degree, illustrated the importance of monitoring what her 2-year-old child did with the device in response to the question about mobile screen technologies being bad for children’s learning, “*no si tu estas al pendiente de, de lo que el esta mirando*.” [English translation: “*not if you are aware of, of what he is watching*”].

#### Co-use

Finally, the fifth type of mediation strategy that was only mentioned by a fifth of the parents (20%) stressed the importance of co-using the mobile device with the child in order to assist them or to help them understand the content they were viewing or using. Although fewer parents mentioned this mediation strategy as being important for children’s learning, the parents who did mention it were distributed equally across income (*n* = 4 low, *n* = 5 middle-to-high income), gender (*n* = 5 mothers, *n* = 4 fathers), and linguistic groups (*n* = 2 monolingual Spanish-speaking, *n* = 6 English-Spanish bilingual, 1 = monolingual English-speaking). In contrast with patterns from previous mediation strategies, however, only parents with a high school degree or more discussed the importance of co-use for their child’s learning. For example, Luis, a middle-to-high income English-Spanish bilingual father with a Master’s degree talked about an experience when his son asked him a question about the show he was viewing on his tablet, “*so my son is learning about the brain, so because I know that he’s watching the Magic School Bus, I’ll say, yes son. You go in through the nose and did you see that they went and they got, and they learned about the brain’s connections, and that the brain has all these connections, right? And that the brain has all these capacities, right? So he is learning, right? But that learning is not happening if I’m not closing those gaps, right?*”

#### Combination of Mediation Strategies

In addition to most parents talking about the importance of using some form of mediation strategy to ensure children benefited from mobile screen technologies, slightly more than half of the parents (52%) also mentioned the importance of using multiple types of mediation strategies. Notably, these parents were spread across income (*n* = 9 low, *n* = 12 middle-to-high income), gender (*n* = 12 mothers, *n* = 9 fathers), and linguistic groups (*n* = 4 monolingual Spanish-speaking, *n* = 16 English-Spanish bilingual, 1 = monolingual English-speaking), as well as education levels (elementary school-Ph.D.). Nevertheless, it should be noted that more middle-to-high income mothers (*n* = 8) discussed the importance of using a combination of mediation strategies than middle-to-high income fathers (*n* = 4) and low-income parents (*n* = 4 mothers, *n* = 5 fathers). In the following excerpt, Jennifer, a middle-to-high income English-Spanish bilingual mother who had completed some college, talked about the importance of using several types of mediation strategies (i.e., appropriate content, time limits) with her daughter, “*it just depends how the parents um, how long they let their child use it and what they’re doing with it.*” Similarly, Leslie, a bilingual middle-to-high income mother with a master’s degree mentioned using the mediation strategies of monitoring content and setting time limits for her child, “*of course, I check his videos before so it’s very, you know, talks about friendship. And I set limits to that… I went to the APA and took what they recommended for children on screen time. He has 30 min for educational videos and 30 min for activities*.” Likewise, Luis, a middle-to-high income bilingual father with a Master’s degree, also mentioned the strategies of monitoring, content, and time limits when he stated, “*we’re very aware of the game apps. Like my daughter for example, downloaded an app, like two so she’s played on apps. But again, it’s not an everyday thing. She might do it once every other week for about 20 min.”* It is important to remember that Luis also mentioned co-using the device with his children in the previous section. Thus, Luis engaged in the mediation strategies of monitoring, ensuring content was appropriate, setting time limits, and co-using the device with his children.

In sum, most parents (85%) across income, gender, linguistic groups, and education levels viewed parental mediation strategies as the key factor in determining whether mobile screen technologies benefited or hindered their children’s learning.

## Discussion

This study investigated diverse Latine parents’ beliefs and attitudes about the ways mobile screen technologies supported and/or hindered their young children’s learning and development (ages 0–4). For the most part, our findings showed that parents across income levels, gender, linguistic groups, and education levels thought that they, as parents, played a key role in determining the extent to which mobile screen technologies positively or negatively influenced their children’s learning, and only minor differences were noted across groups.

In general, parents thought that by using mediation strategies, such as ensuring their children viewed appropriate content, setting time limits for their children’s use of devices, and continuously monitoring their children while they used a device, they could ensure that their children primarily benefited from using mobile screen devices. Although research exploring parental mediation strategies in the context of mobile screen technologies is still limited, the forms of mediation practices parents in our study described using are consistent with those found in the limited but growing body of research on mobile screen technologies and young children (e.g., Beyens and Beullens, 2017; Tang et al., 2018; Domoff et al., 2019), and those found in the extensive research on TV, which have been primarily been conducted among middle-class, White parents (Nathanson, 1999, 2001; Warren, 2003; Collier et al., 2016; Piotrowski, 2017). Furthermore, the diverse Latine parents in this study described being cognizant of the important role they play in mediating their children’s use of mobile screen devices. This is particularly meaningful since extensive research in the context of TV has found that the types of mediation strategies parents engage in are related to children’s success in learning from screens (Nathanson, 1999; Livingstone et al., 2015). Specifically, viewing age-appropriate and educational content has been associated with children’s letter recognition, numeric skills, vocabulary, behavior, and cognitive scores (Linebarger and Walker, 2005; Tomopoulos et al., 2010).

However, despite finding that most mediation strategies were spread across groups, we did note two differences. First, almost a quarter of the parents with a high school degree or more stressed the importance of actively co-using devices with their children to ensure their child knew how to use the device and also understood the content. Second, more educationally diverse, low-income parents than middle-to-high income parents and more fathers than mothers mentioned the importance of continuously monitoring their children’s use of devices so that they did not encounter inappropriate content. Other research has found that parents with lower incomes have less knowledge about technology and privacy-protecting features than higher income parents (Nikken and Opree, 2018), and that lower income parents utilize more free, commercial-laden apps as part of the “app economy” (Burroughs, 2017). This could help explain these patterns, if differences exist in access to information and high-quality and commercial-free apps between families with more and fewer financial resources, then children from lower-income homes might be at a greater risk of encountering third-party advertisements and having fewer parental control settings, which would explain why lower-income parents in our sample stressed the importance of more hands-on monitoring during use. Finding that more fathers than mothers mentioned the importance of continuously monitoring their children while using the mobile screen device is a new finding that has not been explored in previous research. In our sample, fathers were slightly older than mothers. Thus, it could be that older parents considered continuously monitoring their children more important than younger parents.

Our finding of income differences in mediation is consistent with the mediation of TV literature, which finds that middle-to-high income parents are more likely to endorse active co-use of the TV than low income parents (Warren, 2003), and that low income parents are more likely to endorse more restrictive forms of mediation than middle-to-high income parents (Warren, 2003). These differences are meaningful because past research on TV has shown that viewing appropriate content and active co-use of devices are two of the most effective mediation strategies in ensuring that children learn from screens. Specifically, co-viewing TV and co-using mobile screen devices have been found to be among the most effective mediation strategies in promoting child learning, especially among young children (Zack and Barr, 2016; Herodotou, 2017; Sheehan et al., 2019). This is because parents can use this time to help their child better navigate the device and/or understand the concepts they are viewing or reading about through the use of relevant and appropriate scaffolds, such as explaining or elaborating in a way the child can understand (Zack and Barr, 2016). In fact, research among toddlers has shown that children can transfer learning from screen devices to real life when their parents engage in high quality interactions while they co-use the device (Zack and Barr, 2016).

More than income differences, formal education seems to matter to utilizing the mediation strategy of co-use. We found that parents with a high school education or higher more often described engaging in active co-use of devices to ensure their children learned, compared to parents with less educational attainment. Thus, efforts might need to be made to reach parents with lower levels of formal education (i.e., less than high school diploma) and provide them with information about the benefits of actively co-using mobile devices with their children along with specific tips on how to actively co-use devices (Zack and Barr, 2016). In providing this information, researchers should also stress the importance of actively engaging with the child while they use the device as opposed to just passively sitting next to the child, but not engaging in discussions or conversations (i.e., active versus passive co-use).

In addition to expressing the importance of implementing mediation strategies, all parents in our study believed that mobile devices could benefit their children by helping them learn concepts or develop their language skills. The lack of differences in this belief between low-income and middle-to-high income parents is in contrast with most of the existing literature, which finds that low-income parents are more likely than middle-to-high income parents to attribute learning benefits to mobile screen technologies (Rideout, 2014; Radesky et al., 2016). Furthermore, when asked about the ways mobile screen technologies negatively affected their children’s learning, a large portion of parents across income, education levels, gender, and language groups talked about the risk of being exposed to inappropriate content. Noticeably, none of the parents in our sample talked about purchasing apps or subscriptions to reduce the pop-up ads their children were exposed to while viewing Youtube videos or using apps. Furthermore, only six parents, with a high school degree or more, mentioned having content restrictions on the device to control the content their children were exposed to (e.g., Youtube for children). This suggests that more efforts should be made toward making apps and videos targeted toward young children ad-free and providing guidance for parents on how to utilize parental controls.

Parents also talked about the negative effect mobile devices could have on their children’s social interactions and about the danger of becoming dependent on the device. These concerns are similar to the views expressed by ethnically diverse parents in survey studies and the few interview studies on mobile screen technologies (Wartella et al., 2014; Radesky et al., 2016; Common Sense Media, 2017; Sergi et al., 2017; McCloskey et al., 2018). Future efforts should provide parents with information and tips on how to reduce the risk of device dependence and ways to recognize signs of device addiction. Importantly, researchers should be cognizant of parents’ financial situation when recommending alternative activities to device use. Ideally, the alternatives should be free and easily accessible to parents across the income spectrum, and also feasible for parents who are tired from working long shifts.

Parents, for the most part, viewed mobile devices as having the potential to be beneficial to their children’s learning, but their control through the use of mediation practices, determined the benefit as well as risk. This is promising for future media interventions that could build on parents’ existing views about mediation practices and help bolster optimal practices. In other words, parents are already aware that they play a vital role in determining whether mobile screen technologies have a positive or negative effect on their children’s learning. As such, interventions should capitalize on this awareness and focus on increasing parents’ knowledge about effective mediation strategies, particularly active co-use of mobile devices and time limits, especially for younger children.

### Limitation

There are a few limitations worth mentioning. First, although we obtained an equal number of monolingual Spanish-speaking mothers (*n* = 3) and fathers (*n* = 3), they were all from lower income families with very low formal educational attainment. Additionally, the vast majority of the sample spoke English (*n* = 34), although 33 were bilingual. Hence, we were not able to capture how experiences may differ between monolingual Spanish-speaking and monolingual English-speaking parents, especially across different economic and educational backgrounds. Because language and SES were confounded for the small sample of Spanish-speaking parents, it was difficult to discern whether some of the findings were attributable to their language, which is often used as a proxy for acculturation, or their education level. Moreover, only one monolingual Spanish-speaking mother had a bachelor’s degree. Future studies should place more effort toward obtaining a more socioeconomically diverse sample of Spanish-only speaking parents.

Secondly, a large portion of the low-income fathers (40%) and mothers (60%) in our sample were born in Mexico and Ecuador compared to the majority of middle-to-high income mothers (90%) and fathers (90%) who were born in the United States. Given that research in other topics about parenting beliefs has found that foreign-born Latina mothers sometimes conceptualize parenting topics differently from US-born Latina mothers (Zepeda and Espinosa, 1988), it is possible that we did not fully capture the experiences of low-income, US-born Latina mothers. Nevertheless, most of our findings appeared to be driven by education level and gender, rather than country of origin.

Our sample was unexpectedly and primarily composed of parents who were married or living with their partner. Hence our findings might not generalize to single parents. Additionally, the majority of the parents in our sample happened to be parents to sons. Thus, it is possible that patterns might be different for parents of daughters. Future studies should include parents with equitable numbers of sons and daughters. Finally, it is also important to mention that we only examined parent beliefs about the role of mobile devices on their children’s learning and not actual practices. Therefore, it is possible that beliefs might not always translate to actual practices for some parents. This underscores the need for future work to examine whether parent beliefs about their role as parents in mediating their children’s experiences with mobile devices are related to their actual mediating practices.

## Conclusion

This study addressed an important gap in the literature by investigating how socioeconomically and linguistically diverse Latine mothers and fathers believed mobile screen technologies benefit and/or hinder their children’s learning. Our findings suggest that low and middle-to-high income mothers and fathers with diverse levels of education and linguistic abilities are well aware of the important role they play in mediating their children’s use of mobile devices to benefit their learning and protect against potential harms. These findings also underscore the importance of not just including diverse ethnic groups but also considering the heterogeneity within ethnic groups. Observed differences based on gender, income, language and education are important and indicate that guidance around mobile screen device use could be tailored for different types of parents. These findings can help inform future work that seeks to promote optimal media habits among diverse Latine families. Importantly, because there are more similarities than differences across groups, it enables many intervention efforts and information resources to look similar, with other materials being tailored such as targeted materials for parents with little formal education (e.g., less than high school diploma).

Regardless of income or ethnicity, mobile devices are part of almost all young children’s lives (Common Sense Media, 2017). However, parental beliefs about the way these devices can support their children’s learning and the ways in which parents can bolster the benefits and minimize detriments have not been well studied across different racial and ethnic groups. This study demonstrates the many similarities and few differences in beliefs and practices among socioeconomically diverse mothers and fathers within the same ethnic group. To better understand how these ever-present devices relate to young children’s learning, research should include more educationally, economically, racially, and ethnically diverse families.

## Data Availability Statement

The original contributions presented in the study are included in the article/supplementary material, further inquiries can be directed to the corresponding author.

## Ethics Statement

The studies involving human participants were reviewed and approved by University of California, Irvine IRB Committee. The participants provided their written informed consent to participate in this study.

## Author Contributions

WO the first author thought about the research idea, collected the data, analyzed the data, and wrote the manuscript. SR the second author supported the first author in conceptualizing and polishing the research idea along with the methodology, helped with developing and verifying the codes, wrote portions of the manuscript, and helped edit the final draft. Both authors contributed to the article and approved the submitted version.

## Conflict of Interest

The authors declare that the research was conducted in the absence of any commercial or financial relationships that could be construed as a potential conflict of interest.

## Footnotes

1 The term Latine is used to refer to individuals whose cultural background originated in Latin America. In United States academic circles, Latinx is used as a gender-inclusive term to refer to people from Latin American backgrounds. Many Spanish-speakers, including the first author of the current study – a native Spanish-speaker, find the term unpronounceable (see Zentella, 2017). Thus, we have opted to use the gender-inclusive term, Latine, commonly used throughout Spanish-speaking Latin American countries.
